# Bioactive Peptides in Cancer: Therapeutic Use and Delivery Strategies

**DOI:** 10.1155/2013/568953

**Published:** 2013-05-15

**Authors:** Paola Stiuso, Michele Caraglia, Giuseppe De Rosa, Antonio Giordano

**Affiliations:** ^1^Department of Biochemistry Biophysics and General Pathology, Second University of Naples, Via L. De Crecchio 7, 80138 Napoli, Italy; ^2^Department of Pharmacy, University of Naples Federico II, Via Montesano, 80131 Naples, Italy; ^3^Sbarro Institute for Cancer Research and Molecular Medicine Temple University Philadelphia, PA 19122, USA

Human cancer is one of the most important causes of death in western and industrialized countries. In advanced stages, therapeutic opportunities are still limited due to the difficulty to target specifically only cancer cells sparing healthy ones. Cancer often has specific molecular alterations of signal transduction pathway or of other molecules linked to the proliferation and wide spreading of tumour cells. Several peptides have been isolated from natural products and/or physiological sources and several of them have biological activity on cancer cells. This special issue addresses the role of these natural peptides and of their synthetic derivatives in cancer therapy, alone or in combination with other anticancer agents. 

One of the most interesting peptides of natural origin is Urotensin-II (U-II) originally isolated from the goby urophysis and subsequently identified in humans. It binds a specific receptor (UTR) that mediates a very strong contraction of vessel muscle cells. In particular, since the modulation of the U-II system offers a great potential for therapeutic strategies related to the treatment of several diseases, like cardiovascular diseases, the research of selective and potent ligands at UTR is more fascinating. Finally, it was recently reported by several groups a potential role of U-II and its receptor in the regulation of cancer biology. The paper by F. Merlino et al. discusses peptide and nonpeptide U-II and UTR structures that have led to a more rational and detectable design and synthesis of new molecules with high affinity for UTR. Other peptides with anticancer properties are peptide hormones acting on the pituitary axis in the treatment of endocrinological cancers such as breast and prostate cancer and peptides acting on neuroendocrine receptors such as somatostatin and BN/GRP (bombesin/gastrin-releasing peptide). The paper by J. Thundimadathil discussed the role of luteinizing hormone-releasing hormone (LHRH) agonists and antagonists and on the possible action of somatostatin analogues as radioisotope or cytotoxic drug carriers in the treatment of somatostatin receptor expressing tumours. Finally, the possible use of peptides in cancer vaccine or anti-angiogenic strategies is also described. 

Food is another important source for proteins and peptides with potential anticancer activity. Bovine milk is an important component of the human diet, and it contains several proteins formed by two major families: caseins (insoluble) and whey proteins (soluble). The latter are *β*-lactoglobulin, *α*-lactalbumin, bovine serum albumin, and lactoferrin while the predominant forms of the caseins in ruminant milk are *α*S1, *α*S2, *β*, and *κ*. The paper by Pepe et al. describes the main evidences on the anticancer activity of some of these proteins on human cancer cell cultures. Another important function of certain peptides in cancer cell biology is the potential interference with the oxidative processes that are, in turn, strictly implicated in the occurrence and progression of cancer. In this light, cyclooxygenase (COX) is a key enzyme in the biosynthetic pathway leading to the formation of prostaglandins, which are mediators of inflammation and oxidative stress. It exists mainly in two isoforms COX-1 and COX-2, with the latter being more expressed in cancer cells than in normal counterparts. Therefore, agents that inhibit COX-2 while sparing COX-1 represent a new attractive therapeutic development and offer a new perspective for a further use of COX-2 inhibitors. The paper by Vernieri et al. describes the design of new tripeptide inhibitors of COX-2 with, in some cases, a potent and selective inhibitory activity of the enzymatic function of COX-2. These peptides are, therefore, promising as anticancer agents. Another peptide that has demonstrated to be a strong modulator of the stress response is a quinone-based mimetic dipeptide, named DTNQ-Pro. It has been previously reported to induce differentiation of growing Caco-2 cells through inhibition of heat shock proteins (HSPs) 70 and 90. The paper by Gomez-Monterrey et al. has evaluated whether a decrease of stress proteins induced by DTNQ-Pro in Caco-2 cells could sensitize them to treatment with 5-fluorouracil (5-FU). The pretreatment of Caco-2 with DTNQ-Pro increases lipid peroxidation and decreases expression of p38 mitogen-activated protein kinase (MAPK) and FOXO3a. At the same experimental conditions, an increase of the 5-FU-induced growth inhibition of Caco2 cells was recorded. These effects could be due to enhanced DTNQ-Pro-induced membrane lipid peroxidation that, in turn, causes the sensitization of cancer cells to the cytotoxicity mediated by 5-FU. Again the modulation of oxidative stress by a peptide could be a way to sensitize cancer cells to cytotoxic antitumour agents such as 5-FU. Similarly, the second-generation peptide (CIGB-552) described in the paper by Fernández Massò et al. increases the levels of COMMD1, a protein involved in copper homeostasis, sodium transport, and NF-*κ*B signaling pathway. These effects were recorded together with the decrease of the antioxidant capacity of cancer cells paralleled by proteins and lipids peroxidation. This study provides new insights into the mechanism of action of the peptide CIGB-552, which could be relevant in the design of future anticancer therapies. 

Tumour microenvironment is becoming even more important in the regulation and sustainment of cancer cell growth. In this light, matrix metalloproteinases (MMPs) are important medicinal targets for cancer invasion and metastasis, where they showed to have a dual role, inhibiting or promoting important processes involved in the pathology. MMPs contain a zinc (II) ion in the protein active site. Tortorella et al. have designed small-molecule inhibitors of these metalloproteins that bind directly to the active site containing metal ions. In this paper, they describe the synthesis and preliminary biological evaluation of amino acid derivatives as new zinc binding groups (ZBGs). The incorporation of selected metal-binding functions in more complex biphenyl sulfonamide moieties allows the identification of a compound able to interact selectively with different MMP enzymatic isoforms. These results disclose new potential strategies to inhibit cancer metastasization processes.

In conclusion, the present special issue represents, in our opinion, an exciting, insightful observation into the state of the art, as well as emerging future topics, in this important interdisciplinary field. Peptides and proteins of natural sources and their synthetic derivatives can represent an important armamentarium with still unexplored potentials in the treatment of human cancers. The research in this specific field is strongly warranted in order to optimize cancer therapy and in order to increase the hopefully requests of the cancer patients.

*Paola Stiuso* *Paola Stiuso* *Michele Caraglia* *Michele Caraglia* *Giuseppe De Rosa* *Giuseppe De Rosa* *Antonio Giordano* *Antonio Giordano*

